# Not All Plants Are Equal: Diet Quality and Inflammation in Vegans and Vegetarians in Urban Poland

**DOI:** 10.3390/nu17213361

**Published:** 2025-10-25

**Authors:** Martyna Mrozik, Oliwia Grygorczuk, Anna Lipert, Adam Białas, Sylwia Kamińska, Wojciech Drygas, Ewa Rębowska, Stanisław Łegocki, Anna Jegier, Katarzyna Szmigielska, Magdalena Kwaśniewska

**Affiliations:** 1 Department of Preventive Medicine, Medical University of Lodz, 90-752 Lodz, Poland; anna.lipert@umed.lodz.pl (A.L.); wojciech.drygas@umed.lodz.pl (W.D.); ewa.rebowska@umed.lodz.pl (E.R.); stanislaw.legocki@umed.lodz.pl (S.Ł.); magdalena.kwasniewska@umed.lodz.pl (M.K.); 2 Lifestyle Medicine Students’ Scientific Association at the Department of Preventive Medicine, Medical University of Lodz, 90-752 Lodz, Poland; oliwia.grygorczuk@student.umed.lodz.pl (O.G.); sylwia.smarzych@student.umed.lodz.pl (S.K.); 3 Department of Pneumology, Medical University of Lodz, 90-419 Lodz, Poland; adam.bialas@umed.lodz.pl; 4 Department of Sports Medicine, Medical University of Lodz, 92-213 Lodz, Poland; anna.jegier@umed.lodz.pl (A.J.); katarzyna.szmigielska@umed.lodz.pl (K.S.)

**Keywords:** plant-based diet, diet indices, Healthful Plant-Based Diet Index, chronic low-grade inflammation, vegans, vegetarians, nutritional epidemiology

## Abstract

**Background**: Accumulating evidence suggests that dietary factors may affect cardiometabolic health, but the associations between the quality of plant-based diets and chronic-low grade inflammation have been insufficiently explored. The aim of this study was to examine the association between dietary indices and inflammatory markers in the studied cohort of vegans and vegetarians living in urban Poland. **Methods:** The study population comprised 198 individuals (mean age 33.6 yrs) including vegans (VG; *n* = 50), vegetarians (VN; *n* = 101) and omnivores (OV; *n* = 47). The following methods were used in this analysis: a questionnaire interview, anthropometric measurements and blood sample collection. Dietary patterns were evaluated using the Food Frequency Questionnaire and overall plant-based diet index (PDI) and healthful plant-based index (hPDI) were used to define adherence to plant-based dietary patterns. **Results:** Vegans had substantially lower hsCRP concentration and lymphocyte counts than VN and OV (*p* < 0.05). IL-6 concentrations, as well as total WBC, neutrophils, and lymphocytes counts, were significantly higher in OV compared to the other groups. In the overall study population, higher intake of plant-based foods was associated with lower mean levels of hsCRP, IL-6, glucose, lipids, neutrophil, lymphocyte, and total WBC counts (*p* < 0.05). Among vegetarians, higher consumption of healthful plant-based foods was associated with lower levels of total cholesterol, triglycerides, and selected inflammatory biomarkers. **Conclusions**: Our research indicates that the quality of plant-based diets is a critical determinant of cardiometabolic and inflammatory health. Importantly, eliminating animal products alone does not guarantee health benefits; rather, the composition and quality of plant-based foods are key.

## 1. Introduction

Chronic low-grade inflammation (CLGI) is defined by a persistent, mild elevation of circulating pro-inflammatory cytokines and sustained activation of immune cells [1]. This chronic inflammatory milieu is increasingly recognized as a key pathogenic mechanism driving the development of numerous chronic diseases. Elevated concentrations of inflammatory markers, including C-reactive protein (CRP), interleukin-6 (IL-6), and tumor necrosis factor-alpha, have been consistently linked to cardiovascular disease, cancer, diabetes, obesity, and other metabolic disorders [2,3,4,5,6,7].

Among lifestyle factors, habitual dietary patterns have been identified as major contributors to CLGI [8,9,10,11]. An expanding range of studies suggests that both overall dietary patterns and specific nutrients can profoundly modulate the inflammatory state [12,13,14]. A considerable amount of research indicates that consumption of diets containing high levels of saturated fats and processed red meat, combined with low fiber intake, are associated with an enhanced pro-inflammatory response [15,16,17,18,19]. Conversely, frequent intake of vegetables, fruits, and fish has been consistently related to lower levels of systemic inflammation [20,21]. The Mediterranean diet, which emphasizes plant foods, healthy sources of fat, and moderate fish intake, has demonstrated protective effects against CLGI [18,19,22,23]. In contrast, diets rich in fat and processed foods have been positively associated with elevated circulating biomarkers of chronic inflammation [16,24].

Growing evidence demonstrates that adopting plant-based diets (PBDs) substantially reduces the risk of noncommunicable chronic diseases (NCDs). These dietary patterns emphasize foods derived primarily from plant sources, with varying degrees of restriction or exclusion of animal products. Vegetarian diets, as a subset of PBDs, exclude some or all forms of animal-derived foods, whereas vegan diets eliminate all animal products, including meat, dairy, eggs, fish, and often honey [25].

Globally, PBDs are gaining popularity driven by ethical, environmental, and health-related motivations [26,27,28]. They are typically associated with health-promoting food choices such as vegetables, fruits, legumes, whole grains, nuts, and seeds, consumed in minimally processed forms. Accumulating data indicate protective role of PBDs in reducing the risk of coronary heart disease, various cancers, type 2 diabetes, and other NCDs [29,30,31,32,33,34,35,36,37,38].

Our previous analyses conducted among flexitarians, vegetarians, and vegans demonstrated that reducing the intake of animal-derived products or adhering to a predominantly plant-based diet may favorably influence cardiometabolic health compared with traditional Western dietary patterns [39,40]. The beneficial effects of PBDs on systemic inflammation are thought to derive not only from the overall dietary pattern but also from the presence of specific anti-inflammatory nutrients, including antioxidants and phytochemicals [41,42,43]. In light of accumulating scientific evidence, plant-derived foods have been increasingly emphasized in international nutrition guidelines as a core component of health-promoting dietary strategies [28,44,45,46].

In parallel, the global food industry is responding to evolving consumer preferences and nutritional recommendations by expanding the availability of plant-based products. However, this rapid market growth presents new challenges. An increasing number of ultra-processed plant-based alternatives to meat and dairy have emerged, and these products do not necessarily align with the nutritional quality traditionally associated with whole-food, plant-based diets [47]. This trend raises important questions about the health implications of such products and underscores the need for careful evaluation of their composition and role in long-term dietary interventions.

It is essential to note that not all foods derived from plants are inherently health-promoting. Products such as refined grains, sugar-sweetened beverages, snacks, and confectionery items—often rich in processed fats, added sugars, sweeteners, artificial flavors, or salt—can still be classified as “plant-based” if they are free from animal-derived ingredients [47,48,49]. This underscores a crucial distinction between plant-based diets centered on whole, minimally processed foods and those dominated by ultra-processed plant-derived products.

To improve assessment of plant-based diets, several dietary indices have been introduced. Among the most widely used are the Plant-Based Dietary Index (PDI) and its derivatives—the healthful PDI (hPDI) and the unhealthful PDI– offer a nuanced evaluation of plant food consumption by differentiating between beneficial and less beneficial plant-based foods [50]. These tools provide a more precise evaluation of intake of plant-based foods on a daily basis and help clarify the relationship between plant-based diet composition and health outcomes.

Population-based studies have emphasized that the quality and composition of PBDs are critical determinants of morbidity and mortality [31,35,50,51,52,53]. Findings from the UK Biobank project showed that higher adherence to the hPDI was associated with significantly lower risks of total mortality, cardiovascular disease (CVD), and cancer, including reduced incidence of myocardial infarction and ischemic stroke. Conversely, higher intake of unhealthy plant-based foods were linked to increased risks of mortality, CVD, and cancer among middle-aged adults [54]. Similarly, data from U.S. prospective cohorts revealed that higher hPDI scores were associated with reduced risks of colon and breast cancers [33,55].

Collectively, these findings highlight the importance of distinguishing between health-promoting and less beneficial plant-based foods when evaluating the long-term health effects of PBDs. Among proposed mechanisms underlying the health benefits of healthy plant-based dietary patterns, the reduction of CLGI is considered one of the most plausible pathways [30,52]. However, evidence directly linking plant-based dietary indices to inflammatory biomarkers remains limited—particularly in populations adhering to vegetarian or vegan diets [53,56,57,58]. The study by Kharaty et al. provided compelling evidence that adherence to a more healthful PBD is associated with a more favorable inflammatory profile. Nonetheless, this analysis was conducted in a general population with heterogeneous dietary habits and did not specifically focus on individuals following vegetarian or vegan dietary patterns [56].

Existing studies targeting vegetarian or vegan cohorts have typically assessed only a single inflammatory biomarker—most often high sensitivity (hs)CRP—without incorporating dietary quality indices [56,59,60]. Consequently, there remains a substantial gap in the literature regarding the interplay between plant-based diet quality and systemic inflammation within strictly defined plant-based cohorts.

To address this gap, the present study investigated the association between dietary patterns and inflammatory profiles among Polish adults, with particular emphasis on diet quality as measured by plant-based dietary indices. By comparing inflammatory biomarkers in individuals adhering to plant-based versus omnivorous diets, this analysis sought to elucidate whether the composition and nutritional quality of plant-based diets contribute to the modulation of CLGI.

## 2. Materials and Methods

### 2.1. Study Population

The study was carried out between January 2022 and May 2023 within the Department of Preventive Medicine, the Students’ Scientific Association of Lifestyle Medicine and the Department of Sports Medicine, Medical University of Lodz, Poland. Written informed consent was obtained from all participants prior to their involvement in the study. The study protocol was approved by the Ethics Committee of the Medical University of Lodz (RNN/287/21KE), in accordance with the Declaration of Helsinki. 

Recruitment focused on adults in urban Lodz adhering to plant-based diets for at least 12 months, using posters, leaflets, and social media. Invitations provided information on the study’s objectives and methodology. Participants were scheduled for examinations and received detailed information regarding the procedures, including their indications and contraindications. A detailed description of research procedures, recruitment process, and participant classification has been published previously [40]. Upon completion of the study, participants received their examination results along with individualized recommendations from specialists within the research team.

For the purpose of this analysis the study population comprised 198 individuals (mean age 33.6 years) including 101 vegetarians (mean age 32.9 ± 11.5 years), 50 vegans (mean age 33.3 ± 8.5 years) and 47 omnivores (mean age 35.6 ± 8.2 years).

**Figure 1:**
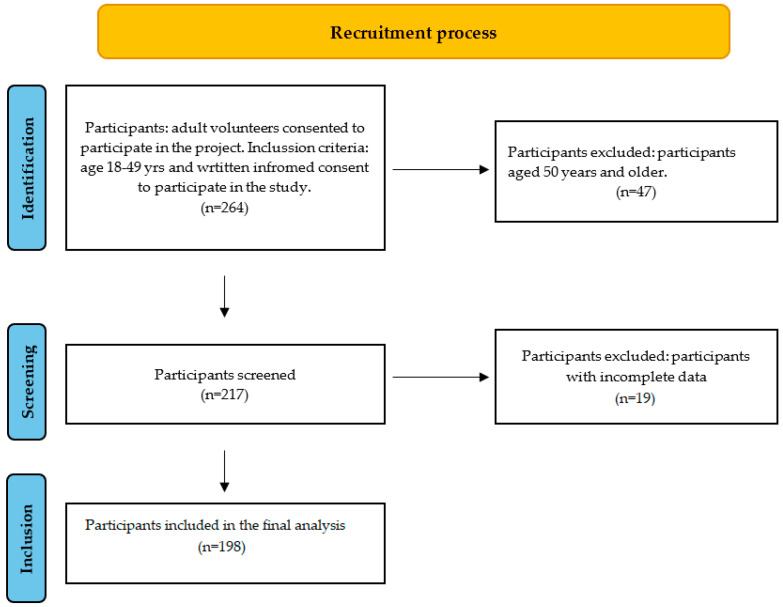
Flow chart of participant recruitment.

Participants were classified according to information obtained from dietary interviews. Vegetarians were defined as individuals who excluded all types of meat (including poultry and fish) but consumed other animal-derived products, whereas vegans excluded all animal products for at least 12 months prior to enrollment. Omnivores were defined as participants who reported any meat consumption, even if they were initially invited as potential vegetarians or vegans. Figure 1 describes the flow of recruitment.

### 2.2. Subjects

#### 2.2.1. Data Collection

Participants were instructed to arrive at the center between 07:30 and 09:00 after at least 12 h of overnight fasting and rest. The study protocol comprised a comprehensive assessment, including questionnaire-based interviews, anthropometric and blood pressure measurements, endothelial function evaluation, and blood sample collection. All the participants completed the questionnaire covering sociodemographic characteristics, smoking status, dietary patterns, physical activity levels, and medical history. The survey was constructed using measures applied in our prior studies [39,40].

#### 2.2.2. Assessment of Lifestyle Characteristics and Inflammation Biomarkers

Anthropometric measurements were performed by qualified practitioners with calibrated instruments. Body weight was measured in kilograms without shoes, to the nearest 100 g, using a calibrated portable electronic Tanita MC-780MA-N S weighing scale (Tanita, Tokyo, Japan). Height was measured in centimeters to the nearest 0.5 cm using a portable stadiometer. BMI, following WHO standards, was calculated by dividing weight in kilograms by the square of height in meters. Waist and hip circumferences were measured in centimeters using a metric tape measure. Body fat, visceral fat, and fat-free mass were obtained from BIA assessment (Tanita MC-780MA-N S)(Tanita, Tokyo, Japan).

All participants had venous blood collected from the antecubital vein in the supine position after a minimum 8-h fast, upon arrival in the morning. The following parameters were evaluated for this analysis: C-reactive protein (hsCRP), IL-6, WBC, neutrophils, lymphocytes, monocytes, eosinophils and basophils. C-reactive protein was measured using the immunoturbidimetric method with Tris buffer and anti-CRP antibodies. IL-6 serum concentrations were measured using the electrochemiluminescence immunoassay method (Cobas e 601 Roche) (Roche Diagnostics, Warsaw, Poland). Fluorescence flow cytometry method was used to determine morphotic elements of blood (Sysmex XN-2000) (Sysmex Corporation, Kobe, Japan). The neutrophil-to-lymphocyte ratio (NLR) was calculated as the ratio of neutrophil to lymphocyte counts. Enzymatic methods were used to determine serum total cholesterol, glucose and triglycerides (COBAS INTEGRA 400 Plus, Roche^®^) (Roche Diagnostics, Warsaw, Poland). High-density lipoprotein cholesterol (HDL-C) was measured using the precipitation method. Low-density lipoprotein cholesterol (LDL-C) was calculated using the Friedewald formula (BECKMAN COULTER DxC 700AU) (Beckman Coulter, Inc., Brea, California, USA). The turbidimetric immunoinhibition method was used to determine glycated hemoglobin without the use of manually prepared hemolysate (BECKMAN COULTER DxC 700AU) (Beckman Coulter, Inc., California, USA).

#### 2.2.3. Dietary and Nutrition Assessment

Dietary intake was assessed using a food frequency questionnaire (FFQ) and a 24-h dietary recall. The FFQ evaluated habitual consumption of selected foods over the preceding six months, covering 88 food groups, including meat, fish, dairy, eggs, various fats, vegetables, fruits, beverages, as well as fortified products such as calcium-fortified soy drinks and vitamin B12-fortified soy and oat beverages. Participants reported consumption frequency for each item using six categories: no consumption, less than once a month, once to three times a month, one to three times a week, four to six times a week, or daily. The FFQ was adapted from instruments used in previous national studies [39]. Usual dietary supplementation was also recorded.

Daily food intake was estimated using a 24-h dietary recall, in which participants reported all meals, beverages, and additional food items consumed during the 24 h preceding the study. Foods were categorized by type and origin, and daily energy and nutrient intakes were calculated using Dieta 6.0 software (Institute of Food and Nutrition, Warsaw, Poland, 2018) and standard calorie databases. Analysis of the FFQ and 24-h recall indicated that vegans reported no consumption of meat or other animal-derived products. Vegetarians abstained from meat, including poultry and fish, but consumed other animal products such as eggs, dairy, and honey. Omnivores consumed meat and other animal products with varying frequency, ranging from less than once per month to daily.

According to the analysis of the FFQ and 24-h dietary recall vegans reported no intake of meat or other animal-derived products. Vegetarians reported no consumption of meat (including poultry and fish), but did consume other animal products such as eggs, dairy, and honey. Omnivores consumed meat and other animal products with varying frequencies (from less than once a month to daily).

#### 2.2.4. Plant-Based Diet Indices

Plant-based diet indices (PDIs) were constructed following the method described by Satija et al. [50]. FFQ food items were grouped into 18 categories, which were subsequently classified as ‘healthy’ plant foods (whole grains, fruits, vegetables, nuts, legumes, vegetable oils, and tea/coffee), ‘unhealthy’ plant foods (fruit juices, refined grains, potatoes and salty foods, sugar-sweetened and artificially sweetened beverages, sweets, and desserts), and animal foods (animal fats, dairy, eggs, fish/seafood, meat, and other animal-derived products). The potato group was modified to include pickled and marinated vegetables commonly consumed in Poland. Margarine and alcohol were excluded from the analysis.

Frequencies of consumption of each food were converted into servings consumed per day. Conversion factors for frequencies of intake reported in the food frequency questionnaires were presented in Supplementary Table S1 [61]. The number of servings of foods that belonged to each of the 18 food groups were then summed. The 18 food groups were created on the basis of nutrient and culinary similarities, within larger categories of animal foods and healthy and less healthy plant foods. Each food group was categorized as ‘positive’ (higher intake indicating higher adherence) or ‘reverse’ (higher intake indicating lower adherence) [50]. Detailed scoring is presented in Supplementary Table S2.

For the overall PDI, participants in the highest tertile of plant-based food intake and lowest tertile of animal-based food intake received a score of 3, whereas those in the lowest plant-based and highest animal-based tertiles received a score of 1. For the hPDI, only healthy plant foods received positive scores. Participants with the highest consumption of healthy plant-based foods received a score of 3, while participants with the highest consumption of unhealthy plant-based foods and animal-based foods received a score of 1 [50].

The food groups were summarized to generate PDI and hPDI scores. The PDI and hPDI ranged theoretically from 18 to 54, with higher scores indicating greater adherence. In this sample the ranges for each score were 20–52 for the PDI, 23–52 for the hPDI. Examples of foods included in each group are provided in Table 1.

### 2.3. Data Analysis

Statistical analysis was conducted using Statistica version 13.1 (StatSoftPolska Sp. z o.o., 2024 update; www.statsoft.pl (accessed on 1 January 2024)). Variable distributions were assessed with the Shapiro–Wilk test. Due to the fact that the distribution of the studied variables deviated from the normal, differences between groups were evaluated using the Mann–Whitney U test for two independent groups or Kruskal–Wallis ANOVA with the Bonferroni correction used to adjust *p*-values in post hoc tests for more than two groups. Spearman’s correlation coefficient was applied to examine the relationship between diet type and inflammation. The effect size was expressed as Cohen’s d value, calculated as the difference between two means divided by a standard deviation for the data. Effect sizes were interpreted as small (d = 0.20–0.49), medium (d = 0.51–0.79), and large (d ≥ 0.80). A post hoc power analysis was performed using Statistica. Assuming a medium effect size (Cohen’s *d* = 0.5), α = 0.05, and *n* = 198, the achieved statistical power was 0.89, indicating that the sample size was adequate to detect meaningful effects. For all analyses, significant differences were accepted at the level of *p* < 0.05.

## 3. Results

Sociodemographic characteristics of the studied groups have been presented in detail in our previous paper [40]. The majority of vegans and vegetarians were single and had a university degree. Among omnivores, singles and those married or in partnership were present in similar proportions. More than two-thirds of participants had attained higher education, while approximately 25% had secondary education. Most participants across all groups reported moderate or high levels of physical activity

### 3.1. Analysis of Nutrition and Dietary Indexes According to the Dietary Patterns

Table 1 presents the daily intake of each food group across the studied groups. Overall, vegans reported the highest intake of healthful plant foods and the lowest (though not statistically significant) intake of less healthful plant foods compared with the other groups. Statistically significant differences were observed in the consumption of vegetables, nuts, and legumes (healthful plant foods), as well as refined grains and sweets and desserts (less healthful plant foods) (*p* < 0.05). Omnivores generally had the lowest intake of healthy foods, whereas vegetarians declared the highest intake of refined grains. Several significant differences were also observed between vegans and vegetarians in the consumption of both healthful and less healthful plant foods. Analysis of dietary indices revealed significantly higher scores for both the overall Plant-Based Diet Index (PDI) and the hPDI among vegans as compared with the other groups (Table 1).

### 3.2. Inflammatory Profiles According to the Dietary Patterns

Table 2 presents inflammatory biomarkers across the studied groups according to dietary patterns. Vegans had substantially lower hsCRP concentrations than VN and OV (*p*< 0.05). Elevated hsCRP was more frequently observed among OV than in VG and VN. Similarly, IL-6 concentrations, as well as total WBC, neutrophils, and lymphocytes counts, were significantly higher in OV compared to the other groups. Within plant-based groups, vegans showed significantly lower hsCRP levels and lymphocyte counts compared with vegetarians (*p* < 0.05) (Table 2). Other cardiometabolic characteristics, including glucose, lipid profile, blood pressure, and anthropometric measures in VG, VN and OV have been reported in detail as previously mentioned [40].

Further analysis of the relationships between inflammatory biomarkers and other cardiovascular characteristics according to plant-based dietary indices is presented in Table 3. Several statistically significant negative correlations were observed both for PDI and hPDI in relation to inflammatory and cardiovascular parameters (*p* < 0.05). In the overall study population, higher intake of plant-based foods was associated with lower mean levels of hsCRP, IL-6, glucose, lipids, as well as neutrophil, lymphocyte, and total WBC counts. Similar associations were observed for higher consumption of healthful plant-based foods (Table 3).

When stratified into three dietary models, further correlations between the analyzed variables (including inflammatory biomarkers) and dietary scores were observed. Statistically significant associations were noted for several variables within the vegan, vegetarian, and omnivore groups. Among vegetarians, higher consumption of healthful plant-based foods was associated with lower levels of total cholesterol, triglycerides, and selected inflammatory biomarkers. In omnivores, greater intake of plant-based foods correlated with lower glucose, total cholesterol, and LDL-cholesterol levels. Importantly, plant-based food consumption was significantly associated with BMI, particularly among vegans and omnivores (Table 4).

## 4. Discussion

The findings demonstrated that diet quality, as measured by plant-based dietary indices, was significantly associated with selected inflammatory and cardiometabolic profiles among adults in an urban Polish population. Vegans reported the highest consumption of healthful plant-based foods and exhibited the most favorable results, including lower levels of hsCRP, IL-6, lymphocytes, and more beneficial lipid profiles, compared with vegetarians and omnivores. Importantly, vegetarians were more likely to consume less healthful, processed plant foods and had higher levels of TC, LDL-C, and triglycerides, in line with our previous findings [40]. Omnivores had the least favorable outcomes overall. Across all groups, higher PDI and hPDI scores were negatively associated with hsCRP, IL-6, WBC counts, neutrophils, lymphocytes, glucose, and lipid levels, underscoring the role of plant-based diet quality in modulating CLGI and cardiovascular characteristics.

Appropriate dietary habits are fundamental to supporting health and protecting against chronic conditions. Current dietary guidelines emphasize that following a well-balanced, predominantly plant-based diet with limited processing is an effective strategy to improve medical outcomes, enhance overall well-being, and could contribute to decreased mortality [28,44,45,46,62]. Over the last few decades, more people have embraced plant-based nutrition. Studies in epidemiology demonstrate substantial modifications in dietary patterns, emphasizing the rise of plant-based diets across multiple communities [63,64,65]. However, not all plant-based foods exert favorable health effects, highlighting the importance of evaluating the most frequent dietary choices of individuals following such diets. Most previous studies involving vegetarians and vegans have not considered the quality of plant-based foods or their relationship with health outcomes.

In our latest work, we presented the intake of macro- and micronutrients among vegans and vegetarians, as well as the cardiometabolic benefits of plant foods. However, the quality of plant-based diets required further analysis [40]. The current study provides a detailed analysis of diet quality, assessed using plant-based dietary indices, among vegetarians, vegans, and omnivores residing in a large urban area of Poland. To our knowledge, no previous study in the region has applied a fully structured, academically designed protocol, enabling the assessment of diet quality across different nutritional patterns, and to examine associations linking diet quality, CVD characteristics, and markers of low-grade chronic inflammation.

We evaluated diet quality using plant-based dietary indices. These tools enable a more detailed assessment of plant food consumption than other commonly applied indices, including the Mediterranean diet score or AHEI.

According to our results, eliminating or reducing animal-derived products does not necessarily result in a high-quality dietary pattern. Plant-based diets confer greater benefits when they consist predominantly of high-quality, unprocessed foods. This study demonstrated that the number of servings of plant-based foods—particularly healthful plant-based products—might reduce inflammation and beneficially affect several CVD characteristics. When we analyzed PBD indices, we found significant differences in health outcomes not only between omnivores and plant-based groups, but also between vegans and vegetarians. These differences are particularly important given that the groups were relatively homogeneous in terms of age, physical activity level, smoking and BMI, suggesting that dietary patterns and diet quality were likely the key differentiating factors.

In the present study, the most favorable results regarding CVD and inflammatory markers were observed in vegans, whereas significantly worse results were observed among omnivores. We also identified several associations among plant-based diet indices and clinical outcomes, irrespective of dietary pattern. Overall, the greater the consumption of healthful plant-based foods, the lower the levels of low-grade inflammation and CVD markers. The anti-inflammatory potential of higher-quality plant-based diets may be partially explained by their greater antioxidant capacity, improved gut microbiota composition, and increased SCFA production [41]. In our cohort, vegans reported the highest intake of healthful plant-based foods and had the most beneficial results. Importantly, vegetarians were more likely to consume less healthful, processed plant foods than both vegans and omnivores. Furthermore, they consumed vegetables, nuts, and legumes significantly less frequently than vegans. In our previous article, we also demonstrated that vegetarians had higher levels of TC, LDL-C, and TG compared with vegans [40]. Of note, in the national Polish representative study WOBASZ, we revealed that the dietary choices of flexitarians and vegetarians were far from current recommendations [39].

Our findings are, to some extent, in line with those of previous studies. Results of a prospective study by Kim et al. suggested that considering the quality of plant foods is crucial for the prevention of metabolic disorders among individuals habitually consuming plant-based diets [66]. Similarly, Heianza et al. reported that adherence to healthful plant-based dietary patterns significantly attenuated cardiovascular risk in individuals genetically predisposed to obesity [67]. Analyses from the UK Biobank study also showed that adherence to the hPDI at higher levels correlated with reduced risks of total mortality, cancer, and CVD. Higher hPDI scores were linked to lower risks of myocardial infarction and ischemic stroke [54]. In patients with prediabetes and diabetes, plant food quality also proved important: Zhuang et al. found that low consumption of whole grains and high intake of sugar-sweetened beverages and other inappropriate dietary choices were associated with increased CVD risk [68]. Moreover, a recent long-term prospective study by Xu et al. showed that participants in the highest quintile of the hPDI had decreased risk of all-cause and cause-specific mortality [69]. Our results support these findings, demonstrating that higher intake of plant-based foods—particularly healthful plant-based products—was associated with significantly lower serum glucose, total cholesterol, LDL-cholesterol, and triglycerides.

Several studies have investigated the influence of plant-based diets on low-grade inflammation. NHANES analyses revealed that both PDI and hPDI were associated with lower systemic inflammation, measured by hsCRP [70]. Cheng et al. reported that higher adherence to healthful plant-based diets was linked to lower inflammation and adiposity markers among breast cancer survivors, though only hsCRP was significantly related to dietary scores [58]. An Irish study found that higher hPDI scores were associated with lower concentrations of CRP, IL-6, WBCs, neutrophils, and monocytes [56]. Similar findings were reported by Pourreza et al., who observed that adherence to a healthful plant-based diet is associated with lower level of hs-CRP [71]. Hillesheim et al. also found that higher hPDI scores were linked to lower BMI and fasting glucose [72]. In our analysis, PDI and hPDI were negatively associated with several markers, including hsCRP, IL-6, WBCs, neutrophils, lymphocytes, and eosinophils, aligning with these previous observations.

An important aim of the present study was to assess diet quality among individuals habitually following plant-based diets. We found that animal products were not always replaced with healthful alternatives. Vegans appeared to be more conscious of their dietary choices, showing a more favorable lipid profile and lower hsCRP and lymphocyte counts compared with vegetarians. In a study published in Nutraceuticals Research, discretionary food consumption (including sugar-sweetened beverages) was higher among semi-vegetarians and omnivores compared with vegans, lacto-ovo-vegetarians, and pescovegetarians [73]. Our results similarly suggest that diet quality plays a critical role: among omnivores, higher PDI was associated with lower glucose, lipids, and BMI, while among vegetarians, healthful plant-based foods were significantly associated with lipid levels, lymphocytes, and eosinophils. For vegans, a higher intake of unhealthful plant-based foods may be linked to higher BMI and inflammatory markers.

Few studies have assessed diet quality specifically among vegetarians and vegans, and those that have often used different methodologies. For example, a cross-sectional study analyzing ultra-processed food intake in vegetarian diets using PDIs found that greater avoidance of animal products was associated with higher consumption of ultra-processed foods. The nutritional quality of diets was also related to the degree of animal food avoidance. Higher intake of unhealthy processed products was observed among individuals with shorter diet duration and younger age at onset [74]. In another study Swedish vegans, vegetarians, lacto-ovo-vegetarians and pescatarians had higher intakes of legumes and plant-based meat analogs compared to omnivores, and vegans and lacto-ovo-vegetarians had higher intakes of plant-based dairy substitutes (vs. pescatarians and omnivores) [75]. In prospective cohort study on Asian adults with participation of 1763 vegetarians, higher hPDI scores were obtained by those who followed plant-based diets. Nevertheless, their intake of both healthful and unhealthful plant foods was higher than non-vegetarians [76]. In another study performed in a cohort on Korean adults (who mainly composes plant foods like grains and vegetables) higher intake of less healthy plant-based foods were associated with a higher risk of dyslipidaemia. Therefore, the type of animal foods consumed and the quality of plant foods are crucial for managing lipid disorders [77]. Other studies have suggested that although vegans and vegetarians often have lower inflammatory markers, the role of diet quality has not always been assessed. Additionally, a study in Kansas childcare centers found that the most common vegetarian substitution in institutional menus was cheese, which resulted in higher intakes of calories, fat, saturated fat, calcium, and sodium, and lower overall diet quality [78]. These findings further emphasize that excluding animal products does not necessarily ensure optimal nutritional quality. Results from our study indicate a higher intake of unhealthy plant-based products in the vegetarian group, while the highest intake of healthy plant foods was in those on vegan diet. There may be several possible explanations for these observations. Following a vegetarian diet may be associated with choosing less healthy plant-based meat substitutes instead of less processed protein sources, such as legume. It seems to be the result of a lack of knowledge andskills on how to compose and prepare properly-balanced plant-based meals. On the other hand, in the present study, individuals following a vegan diet had the highest consumption of healthy plant-based products, which may be related not only to greater nutritional knowledge but also to greater restrictions on the choice of prepared foods.

Potential weaknesses of the analysis are cross-sectional design precludes causal inference, and several sources of bias—such as selection bias, recall bias, and dietary misreporting—cannot be excluded. Dietary intake was evaluated using self-reported questionnaires, which may lead to misclassification. The non-random sample limits generalizability. Future studies should therefore involve larger, randomized samples and longitudinal designs.

A key strength of this study is the use of a standardized methodology conducted by trained professionals, combined with a detailed inflammatory profile assessment protocol. To our knowledge, this is one of the first studies in Central and Eastern Europe to evaluate plant-based dietary indices in relation to inflammation and cardiovascular outcomes. Including vegans, vegetarians, and omnivores in a single cohort, and assessing diet with validated tools, provides valuable new insights into the role of diet quality in modulating CLGI and cardiometabolic health. Additionally, this study has analyzed the dietary data not only quantitively but also the quality of diet was analyzed by PDI and hPDI to define adherence to plant-based dietary patterns. Lastly, questionnaires used in the present analysis were standardized for use in research to conduct interviews.

## 5. Conclusions

In conclusion, our findings suggest that the quality of plant-based diets is a critical determinant of cardiometabolic and inflammatory health. Healthful plant-based diets, rich in whole, minimally processed plant foods, were associated with lower systemic inflammation and more favorable cardiovascular profiles. In contrast, unhealthful plant-based diets, dominated by refined and ultra-processed plant foods, were associated with less favorable outcomes. Importantly, eliminating animal products alone does not guarantee health benefits; rather, the composition and quality of plant-based foods are key.

## Figures and Tables

**Table 1:** Daily intakes of food groups among the study population.

	Vegans *n* = 50	Vegetarians *n* = 101	Omnivores *n* = 47
**Healthy plant foods, servings/d**			
Whole grains	1.75 (1.6; 2.1)	1.45 (1.4; 1.8)	1.2 (1.1; 1.9) ^b^**^S^
Fruits	1.43 (1.4; 1.8)	1.36 (1.2; 1.5)	1.36 (1.1; 1.6)
Vegetables	1.54 (1.5; 1.8)	1.28 (1.3; 1.5) ^a^*^S^	1.07 (1.0; 1.4) ^b^***^L, c^*^S^
Nuts	1.46 (1.3; 1.8)	0.67 (0.8; 1.2) ^a^***^M^	0.38 (0.5; 1.0) ^b^***^L^
Legumes	1.58 (1.4; 1.7)	1.07 (1.0; 1.2) ^a^***^M^	0.31 (0.4; 0.7) ^b^***^L, c^***^L^
Vegetable oil	1.29 (1.1; 1.6)	0.89 (0.9; 1.2)	0.77 (0.7;1.1) ^b^**^M^
Tea and coffee	1.36 (1.3; 1.7)	2.0 (1.8; 2.0) ^a^**^M^	1.71 (1.4; 2.0)
**Less healthy plant foods, servings/d**			
Fruit juices	0.14 (0.2; 0.5)	0.14 (0.2; 0.4)	0.09 (0.2; 0.5)
Refined grains	0.93 (0.8; 1.2)	1.78 (1.7; 2.2) ^a^***^L^	1.12 (1.0; 1.4) ^c^***^M^
Potatoes	0.8 (0.8; 1.2)	0.6 (0.6; 0.8)	0.65 (0.6; 1.0)
Sugar-sweetened beverages	0.02 (0.1; 0.2)	0.06 (0.1; 0.3)	0.04 (0.1; 0.2)
Sweets and desserts	0.44 (0.5; 0.9)	0.79 (0.8; 1.1) ^a^**^S^	0.73 (0.8; 1.4)
**Animal foods, servings/d**			
Animal fat	0 (0; 0)	0.07 (0.2; 0.3) ^a^***^L^	0.31 (0.4; 0.6) ^b^***^L, c^**^M^
Dairy	0 (0; 0)	1.58 (1.5; 2.0) ^a^***^L^	1.61 (1.5; 2.3) ^b^***^L^
Eggs	0 (0; 0)	0.31 (0.3; 0.4) ^a^***^L^	0.58 (0.4; 0.7) ^b^***^L^
Fish and seafoods	0 (0; 0)	0.0 (0; 0)	0.16 (0.2; 0.3) ^c^***^L^
Meat	0 (0; 0)	0.0 (0; 0)	0.54 (0.2; 0.3)
Miscellaneous animal foods	0 (0; 0)	0.02 (0; 0)	0.02 (0; 0.1) ^b^**^L^
**Food groups, servings/d**			
Healthy plant foods group	10.65 (10.2; 11.9)	9.18 (8.9; 10.1) ^a^***^M^	6.82 (6.9; 9.1) ^b^***^L, c^*^S^
Less healthy plant foods group	3.14 (2.7; 3.7)	3.89 (3.7; 4.6) ^a^*^M^	3.57 (2.9; 4.1)
Animal foods group	0 (0; 0)	2.25 (0.0; 2.7) ^a^***^L^	3.79 (3.3; 4.6) ^b^***^L, c^***^L^
**Plant-based diet index**			
PDI score	42 (41.7; 44.2)	38 (36.5; 38.4) ^a^**^L^	31 (29.7; 33.2) ^b^***^L, c^***^L^
hPDI score	45 (43.1; 45.4)	38 (35.6; 37.5) ^a^**^L^	31 (30.4; 33.4) ^b^***^L, c^***^L^

Data are shown as medians with 95% confidence intervals (CIs). Statistical significance: ^a^—Vegans vs. Vegetarians; ^b^—Vegans vs. Omnivores; ^c^—Vegetarians vs. Omnivores; * *p* < 0.05; ** *p* < 0.01; *** *p* < 0.001. Effect size determined by Cohen’s d: ^S^—small; ^M^—medium; ^L^—large. Abbreviations: PDI—Overall Plant-Based Diet Index; hPDI—Healthful Plant-Based Diet Index.

**Table 2:** Inflammatory biomarkers across dietary patterns in the study population.

	Vegans	Vegetarians	Omnivores
hsCRP mg/L	0.3 (0.4; 1.1)	0.56 (0.6; 1.9) ^a^*^S^	0.6 (0.8; 4.2) ^b^*^S^
<1, f (%)	42/50 (84)	67/100 (67)	29/46 (63)
1–3, f (%)	6/50 (12)	26/100 (26)	11/46 (24)
>3, f (%)	2/50 (4)	7/100 (7)	6/46 (13) ^b^*^, c^*
IL-6, pg/mL	0.75 (2.2; 3.2)	0.75 (1.1; 1.4)	0.75 (10.7; 16.3)
<1.5 f (%)	38/50 (76)	84/101 (84)	26/46 (57)
≥1.5 f (%)	12/50 (24)	16/101 (16)	20/46 (43) ^b^**^S, c^**^S^
WBC, 10^9^/L	4.67 (1.1; 1.7)	5.3 (1.2; 1.6)	6.14 ± 1.5 ^1 b^***^S, c^*^L^
Neutrophils, 10^9^/L	2.77 (1; 1.4)	2.83 (1.1; 1.4)	3.5 ± 1.9 ^1 b^**^S, c^*^M^
Lymphocytes, 10^9^/L	1.48 ±0.4 ^1^	1.62 (0.4; 0.6) ^a^**^M^	1.91 ± 0.5 ^1 b^***^L^
NLR	1.83 (0.7; 1.1)	1.7 (0.5; 4.2)	1.76 (0.06; 0.9)
Monocytes, 10^9^/L	0.45 (0.1; 0.2)	0.45 (0.1; 0.2)	0.47 ± 0.12 ^1^
Eosinophils, 10^9^/L	0.085 (0.07; 0.1)	0.1 (0.1; 0.2)	0.14 (0.1; 0.1)
Basophils, 10^9^/L	0.03 (0.01; 0.02)	0.03 (0.02; 0.02)	0.04 (0.02; 0.02)

Data are shown as ^1^ means ± SD; in other cases data shown as medians with 95% confidence intervals (CIs). Statistical significance: ^a^—Vegans vs. Vegetarians; ^b^—Vegans vs. Omnivores; ^c^—Vegetarians vs. Omnivores;* *p* < 0.05; ** *p* < 0.01; *** *p* < 0.001. Effect size determined by Cohen’s d: ^S^—small; ^M^—medium; ^L^—large. Abbreviations: hsCRP—high sensitive C-reactive Protein, IL-6—Interleukin 6, WBC—White Blood Cell Count, NLR—Neutrophil to Lymphocyte Ratio.

**Table 3:** Correlations between Inflammatory Biomarkers and Cardiovascular Characteristics According to Plant-Based Dietary Indices.

	Plant-Based Diet Index	Healthy Plant-Based Diet Index
hsCRP mg/L	−0.15 *	−0.18 *
IL-6, pg/mL	−0.18 **	−0.13
WBC, 10^9^/L	−0.24 ***	−0.25 ***
Neutrophils, 10^9^/L	−0.17 *	−0.11
Lymphocytes, 10^9^/L	−0.24 ***	−0.37 ***
NLR	0.03	0.16 *
Monocytes, 10^9^/L	−0.08	−0.13
Eosinophils, 10^9^/L	−0.11	−0.22 **
Basophils, 10^9^/L	−0.04	−0.07
Glucose (mmol/L)	−0.20 **	−0.14 *
TC (mmol/L)	−0.36 ***	−0.31 ***
TG (mmol/L)	−0.14 *	−0.19 **
HDL-C (mmol/L)	−0.06	−0.08
LDL-C (mmol/L)	−0.34 ***	−0.27 ***
HbA1C (%)	−0.13	−0.03
BMI (kg/m^2^)	−0.14	−0.12

Statistical significance: * *p* < 0.05; ** *p* < 0.01; *** *p* < 0.001. Abbreviations: BMI—Body Mass Index; HbA1C—Hemoglobin A1C (glycated haemoglobin); HDL-C—High Density Lipoprotein Cholesterol; hsCRP—high sensitive C-reactive Protein, IL-6—Interleukin 6; LDL-C—Low Density Lipoprotein Cholesterol; NLR—Neutrophil to Lymphocyte Ratio; TC—Total Cholesterol; TG—Triglycerides WBC—White Blood Cell Count.

**Table 4:** Correlations of inflammatory biomarkers and cardiovascular characteristics with plant-based dietary indices and dietary patterns.

	Vegans		Vegetarians		Omnivores	
	PDI	hPDI	PDI	hPDI	PDI	hPDI
hsCRP mg/mL	−0.05	0.03	0.08	−0.14	−0.27	−0.01
IL-6, pg/mL	−0.30 *	−0.03	0.01	−0.04	−0.20	−0.26
WBC, 10^9^/L	−0.34 *	−0.20	−0.03	−0.08	0.05	0.01
Neutrophils, 10^9^/L	−0.22	−0.08	−0.01	0.06	−0.02	0.02
Lymphocytes, 10^9^/L	−0.33 *	−0.26	−0.06	−0.28 **	0.18	−0.03
NLR	0.09	0.13	0.05	0.23 *	−0.10	0.05
Monocytes, 10^9^/L	−0.23	−0.07	0.03	−0.17	−0.09	−0.12
Eosinophils, 10^9^/L	−0.24	−0.28*	−0.01	−0.21 *	0.16	0.09
Basophils, 10^9^/L	−0.58	−0.18	0.08	0.04	0.27	0.33 *
Glucose (mmol/L)	0.14	0.07	−0.11	0.03	−0.36 *	−0.37 *
TC (mmol/L)	−0.24	−0.02	−0.04	−0.22 *	−0.29 *	−0.08
TG (mmol/L)	−0.21	−0.08	0.07	−0.19 *	−0.18	0.09
HDL-C (mmol/L)	−0.07	0.03	0.08	−0.01	−0.16	−0.01
LDL-C (mmol/L)	−0.21	−0.01	−0.12	−0.17	−0.29 *	−0.10
HbA1C (%)	0.05	0.17	−0.11	0.05	−0.18	−0.17
BMI (kg/m^2^)	−0.16	−0.34 *	0.06	0.02	−0.32 *	−0.22

Statistical significance: * *p* < 0.05; ** *p* < 0.01. Abbreviations: BMI—Body Mass Index; HbA1C—Hemoglobin A1C (glycated haemoglobin); HDL-C—High Density Lipoprotein Cholesterol; hPDI—Healthful Plant-Based Diet Index; hsCRP—high sensitive C-reactive Protein; IL-6—Interleukin 6; LDL-C—Low Density Lipoprotein Cholesterol; NLR—Neutrophil to Lymphocyte Ratio; PDI—Overall Plant-Based Diet Index; TC—Total Cholesterol; TG—Triglycerides; WBC—White Blood Cell Count.

## Data Availability

The original contributions presented in this study are included in the article. Further inquiries can be directed to the corresponding author.

## Supplementary Materials

The following supporting information can be downloaded at: https://www.mdpi.com/article/10.3390/nu17213361/s1, Table S1: Conversion factors for intake frequencies reported in the Food Frequency Questionnaire (FFQ), adapted from Fatihah et al. (2015) [61] with minor modifications to align with the frequency categories used in the present study; Table S2. Examples of food items included in each of the 18 food groups.

## Abbreviations

The following abbreviations are used in this manuscript:

BMI	Body Mass Index
CI	Confidence Interval
CLGI	Chronic Low-Grade Inflammation
CVD	Cardiovascular Disease
HbA1C	Hemoglobin A1C (glycated haemoglobin)
HDL-C	High-Density Lipoprotein Cholesterol
hPDI	Healthful Plant-Based Diet Index
hsCRP	high sensitive C-reactive Protein
IL-6	Interleukin-6
LDL-C	Low-Density Lipoprotein Cholesterol
NCDs	Non-Communicable Chronic Diseases
NLR	Neutrophils to Lymphocytes Ratio
OV	Omnivores
PBDs	Plant- Based Diets
PDI	Plant-Based Diet Index
TC	Total Cholesterol
TG	Triglycerides
VG	Vegans
VN	Vegetarians
WBC	White Blood Cell Count

## References

[1] Nogueira Silva Lima M.T., Howsam M., Anton P.M., Delayre-Orthez C., Tessier F.J. (2021). Effect of Advanced Glycation End-Products and Excessive Calorie Intake on Diet-Induced Chronic Low-Grade Inflammation Biomarkers in Murine Models. Nutrients.

[2] Minihane A.M., Vinoy S., Russell W.R., Baka A., Roche H.M., Tuohy K.M., Teeling J.L., Blaak E.E., Fenech M., Vauzour D. (2015). Low-Grade Inflammation, Diet Composition and Health: Current Research Evidence and Its Translation. Br. J. Nutr..

[3] Furman D., Campisi J., Verdin E., Carrera-Bastos P., Targ S., Franceschi C., Ferrucci L., Gilroy D.W., Fasano A., Miller G.W. (2019). Chronic Inflammation in the Etiology of Disease across the Life Span. Nat. Med..

[4] Markozannes G., Koutsioumpa C., Cividini S., Monori G., Tsilidis K.K., Kretsavos N., Theodoratou E., Gill D., Ioannidis J.P., Tzoulaki I. (2021). Global Assessment of C-Reactive Protein and Health-Related Outcomes: An Umbrella Review of Evidence from Observational Studies and Mendelian Randomization Studies. Eur. J. Epidemiol..

[5] Luan Y.-Y., Yao Y.-M. (2018). The Clinical Significance and Potential Role of C-Reactive Protein in Chronic Inflammatory and Neurodegenerative Diseases. Front. Immunol..

[6] Gasecka A., Siwik D., Gajewska M., Jaguszewski M.J., Mazurek T., Filipiak K.J., Postuła M., Eyileten C. (2020). Early Biomarkers of Neurodegenerative and Neurovascular Disorders in Diabetes. J. Clin. Med..

[7] Kaptoge S., Di Angelantonio E., Lowe G., Pepys M.B., Thompson S.G., Collins R., Danesh J., Emerging Risk Factors Collaboration (2010). C-Reactive Protein Concentration and Risk of Coronary Heart Disease, Stroke, and Mortality: An Individual Participant Meta-Analysis. Lancet.

[8] Schulze M.B., Martínez-González M.A., Fung T.T., Lichtenstein A.H., Forouhi N.G. (2018). Food Based Dietary Patterns and Chronic Disease Prevention. BMJ.

[9] Bujtor M., Turner A.I., Torres S.J., Esteban-Gonzalo L., Pariante C.M., Borsini A. (2021). Associations of Dietary Intake on Biological Markers of Inflammation in Children and Adolescents: A Systematic Review. Nutrients.

[10] Margină D., Ungurianu A., Purdel C., Tsoukalas D., Sarandi E., Thanasoula M., Tekos F., Mesnage R., Kouretas D., Tsatsakis A. (2020). Chronic Inflammation in the Context of Everyday Life: Dietary Changes as Mitigating Factors. Int. J. Environ. Res. Public Health.

[11] Hart M.J., Torres S.J., McNaughton S.A., Milte C.M. (2021). Dietary Patterns and Associations with Biomarkers of Inflammation in Adults: A Systematic Review of Observational Studies. Nutr. J..

[12] Bonaccio M., Pounis G., Cerletti C., Donati M.B., Iacoviello L., de Gaetano G. (2017). MOLI-SANI Study Investigators Mediterranean Diet, Dietary Polyphenols and Low Grade Inflammation: Results from the MOLI-SANI Study. Br. J. Clin. Pharmacol..

[13] Yahfoufi N., Alsadi N., Jambi M., Matar C. (2018). The Immunomodulatory and Anti-Inflammatory Role of Polyphenols. Nutrients.

[14] Mori T.A., Beilin L.J. (2004). Omega-3 Fatty Acids and Inflammation. Curr. Atheroscler. Rep..

[15] Esposito K., Marfella R., Ciotola M., Di Palo C., Giugliano F., Giugliano G., D’Armiento M., D’Andrea F., Giugliano D. (2004). Effect of a Mediterranean-Style Diet on Endothelial Dysfunction and Markers of Vascular Inflammation in the Metabolic Syndrome: A Randomized Trial. JAMA.

[16] Lopez-Garcia E., Schulze M.B., Fung T.T., Meigs J.B., Rifai N., Manson J.E., Hu F.B. (2004). Major Dietary Patterns Are Related to Plasma Concentrations of Markers of Inflammation and Endothelial Dysfunction. Am. J. Clin. Nutr..

[17] Shiraseb F., Hosseininasab D., Mirzababaei A., Bagheri R., Wong A., Suzuki K., Mirzaei K. (2022). Red, White, and Processed Meat Consumption Related to Inflammatory and Metabolic Biomarkers among Overweight and Obese Women. Front. Nutr..

[18] Tristan Asensi M., Napoletano A., Sofi F., Dinu M. (2023). Low-Grade Inflammation and Ultra-Processed Foods Consumption: A Review. Nutrients.

[19] Ko D.T., Alter D.A., Guo H., Koh M., Lau G., Austin P.C., Booth G.L., Hogg W., Jackevicius C.A., Lee D.S. (2016). High-Density Lipoprotein Cholesterol and Cause-Specific Mortality in Individuals Without Previous Cardiovascular Conditions: The CANHEART Study. J. Am. Coll. Cardiol..

[20] Nettleton J.A., Steffen L.M., Mayer-Davis E.J., Jenny N.S., Jiang R., Herrington D.M., Jacobs D.R. (2006). Dietary Patterns Are Associated with Biochemical Markers of Inflammation and Endothelial Activation in the Multi-Ethnic Study of Atherosclerosis (MESA). Am. J. Clin. Nutr..

[21] Anderson A.L., Harris T.B., Tylavsky F.A., Perry S.E., Houston D.K., Lee J.S., Kanaya A.M., Sahyoun N.R. (2012). Dietary Patterns, Insulin Sensitivity and Inflammation in Older Adults. Eur. J. Clin. Nutr..

[22] Schwingshackl L., Hoffmann G. (2014). Mediterranean Dietary Pattern, Inflammation and Endothelial Function: A Systematic Review and Meta-Analysis of Intervention Trials. Nutr. Metab. Cardiovasc. Dis..

[23] Mena M.-P., Sacanella E., Vazquez-Agell M., Morales M., Fitó M., Escoda R., Serrano-Martínez M., Salas-Salvadó J., Benages N., Casas R. (2009). Inhibition of Circulating Immune Cell Activation: A Molecular Antiinflammatory Effect of the Mediterranean Diet. Am. J. Clin. Nutr..

[24] Nettleton J.A., Matijevic N., Follis J.L., Folsom A.R., Boerwinkle E. (2010). Associations between Dietary Patterns and Flow Cytometry-Measured Biomarkers of Inflammation and Cellular Activation in the Atherosclerosis Risk in Communities (ARIC) Carotid Artery MRI Study. Atherosclerosis.

[25] Islam S.U., Ahmed M.B., Ahsan H., Lee Y.-S. (2021). Recent Molecular Mechanisms and Beneficial Effects of Phytochemicals and Plant-Based Whole Foods in Reducing LDL-C and Preventing Cardiovascular Disease. Antioxidants.

[26] Raptou E., Tsiami A., Negro G., Ghuriani V., Baweja P., Smaoui S., Varzakas T. (2024). Gen Z’s Willingness to Adopt Plant-Based Diets: Empirical Evidence from Greece, India, and the UK. Foods.

[27] Jahn S., Furchheim P., Strässner A.-M. (2021). Plant-Based Meat Alternatives: Motivational Adoption Barriers and Solutions. Sustainability.

[28] Willett W., Rockström J., Loken B., Springmann M., Lang T., Vermeulen S., Garnett T., Tilman D., DeClerck F., Wood A. (2019). Food in the Anthropocene: The EAT-Lancet Commission on Healthy Diets from Sustainable Food Systems. Lancet.

[29] Jafari S., Hezaveh E., Jalilpiran Y., Jayedi A., Wong A., Safaiyan A., Barzegar A. (2022). Plant-Based Diets and Risk of Disease Mortality: A Systematic Review and Meta-Analysis of Cohort Studies. Crit. Rev. Food Sci. Nutr..

[30] Satija A., Hu F.B. (2018). Plant-Based Diets and Cardiovascular Health. Trends Cardiovasc. Med..

[31] Chen Z., Drouin-Chartier J.-P., Li Y., Baden M.Y., Manson J.E., Willett W.C., Voortman T., Hu F.B., Bhupathiraju S.N. (2021). Changes in Plant-Based Diet Indices and Subsequent Risk of Type 2 Diabetes in Women and Men: Three U.S. Prospective Cohorts. Diabetes Care.

[32] Baden M.Y., Shan Z., Wang F., Li Y., Manson J.E., Rimm E.B., Willett W.C., Hu F.B., Rexrode K.M. (2021). Quality of Plant-Based Diet and Risk of Total, Ischemic, and Hemorrhagic Stroke. Neurology.

[33] Romanos-Nanclares A., Willett W.C., Rosner B.A., Collins L.C., Hu F.B., Toledo E., Eliassen A.H. (2021). Healthful and Unhealthful Plant-Based Diets and Risk of Breast Cancer in U.S. Women: Results from the Nurses’ Health Studies. Cancer Epidemiol. Biomarkers Prev..

[34] Dinu M., Abbate R., Gensini G.F., Casini A., Sofi F. (2017). Vegetarian, Vegan Diets and Multiple Health Outcomes: A Systematic Review with Meta-Analysis of Observational Studies. Crit. Rev. Food Sci. Nutr..

[35] Kaiser J., Van Daalen K.R., Thayyil A., Cocco M.T.D.A.R.R., Caputo D., Oliver-Williams C. (2021). A Systematic Review of the Association Between Vegan Diets and Risk of Cardiovascular Disease. J. Nutr..

[36] Jabri A., Kumar A., Verghese E., Alameh A., Kumar A., Khan M.S., Khan S.U., Michos E.D., Kapadia S.R., Reed G.W. (2021). Meta-Analysis of Effect of Vegetarian Diet on Ischemic Heart Disease and All-Cause Mortality. Am. J. Prev. Cardiol..

[37] Papier K., Appleby P.N., Fensom G.K., Knuppel A., Perez-Cornago A., Schmidt J.A., Tong T.Y.N., Key T.J. (2019). Vegetarian Diets and Risk of Hospitalisation or Death with Diabetes in British Adults: Results from the EPIC-Oxford Study. Nutr. Diabetes.

[38] Segovia-Siapco G., Sabaté J. (2019). Health and Sustainability Outcomes of Vegetarian Dietary Patterns: A Revisit of the EPIC-Oxford and the Adventist Health Study-2 Cohorts. Eur. J. Clin. Nutr..

[39] Kwaśniewska M., Pikala M., Grygorczuk O., Waśkiewicz A., Stepaniak U., Pająk A., Kozakiewicz K., Nadrowski P., Zdrojewski T., Puch-Walczak A. (2023). Dietary Antioxidants, Quality of Nutrition and Cardiovascular Characteristics among Omnivores, Flexitarians and Vegetarians in Poland-The Results of Multicenter National Representative Survey WOBASZ. Antioxid..

[40] Grygorczuk O., Mrozik M., Lipert A., Kamińska S., Białas A., Drygas W., Rębowska E., Łęgocki S., Jegier A., Szmigielska K. (2024). Cardiovascular Health and Diet Quality among Vegetarians, Vegans and Omnivores: Insights from a Large Urban Population in Poland. Nutrients.

[41] Aleksandrova K., Koelman L., Rodrigues C.E. (2021). Dietary Patterns and Biomarkers of Oxidative Stress and Inflammation: A Systematic Review of Observational and Intervention Studies. Redox Biol..

[42] Graff E., Vedantam S., Parianos M., Khakoo N., Beiling M., Pearlman M. (2023). Dietary Intake and Systemic Inflammation: Can We Use Food as Medicine?. Curr. Nutr. Rep..

[43] Thomas M.S., Calle M., Fernandez M.L. (2023). Healthy Plant-Based Diets Improve Dyslipidemias, Insulin Resistance, and Inflammation in Metabolic Syndrome. A Narrative Review. Adv. Nutr. Bethesda Md.

[44] Canada H. Canada’s Dietary Guidelines. https://food-guide.canada.ca/en/guidelines/.

[45] Canberra (2013). Australian Dietary Guidelines.

[46] Public Health England in Association with the Welsh Government (2016). Food Standards Scotland and the Food Standards Agency in Northern Ireland Eatwell Guide.

[47] Wickramasinghe K., Breda J., Berdzuli N., Rippin H., Farrand C., Halloran A. (2021). The Shift to Plant-Based Diets: Are We Missing the Point?. Glob. Food Secur..

[48] Martín-Miguélez J.M., Martín I., González-Mohíno A., Souza Olegario L., Peromingo B., Delgado J. (2024). Ultra-Processed Plant-Based Analogs: Addressing the Challenging Journey toward Health and Safety. J. Food Sci..

[49] (2020). Salt Content of Vegan and Plant-Based Food in the out of Home Sector.

[50] Satija A., Bhupathiraju S.N., Rimm E.B., Spiegelman D., Chiuve S.E., Borgi L., Willett W.C., Manson J.E., Sun Q., Hu F.B. (2016). Plant-Based Dietary Patterns and Incidence of Type 2 Diabetes in US Men and Women: Results from Three Prospective Cohort Studies. PLoS Med..

[51] Satija A., Bhupathiraju S.N., Spiegelman D., Chiuve S.E., Manson J.E., Willett W., Rexrode K.M., Rimm E.B., Hu F.B. (2017). Healthful and Unhealthful Plant-Based Diets and the Risk of Coronary Heart Disease in U.S. Adults. J. Am. Coll. Cardiol..

[52] Hemler E.C., Hu F.B. (2019). Plant-Based Diets for Personal, Population, and Planetary Health. Adv. Nutr..

[53] Baden M.Y., Liu G., Satija A., Li Y., Sun Q., Fung T.T., Rimm E.B., Willett W.C., Hu F.B., Bhupathiraju S.N. (2019). Changes in Plant-Based Diet Quality and Total and Cause-Specific Mortality. Circulation.

[54] Thompson A.S., Tresserra-Rimbau A., Karavasiloglou N., Jennings A., Cantwell M., Hill C., Perez-Cornago A., Bondonno N.P., Murphy N., Rohrmann S. (2023). Association of Healthful Plant-Based Diet Adherence With Risk of Mortality and Major Chronic Diseases Among Adults in the UK. JAMA Netw. Open.

[55] Wang F., Ugai T., Haruki K., Wan Y., Akimoto N., Arima K., Zhong R., Twombly T.S., Wu K., Yin K. (2022). Healthy and Unhealthy Plant-based Diets in Relation to the Incidence of Colorectal Cancer Overall and by Molecular Subtypes. Clin. Transl. Med..

[56] Kharaty S., Harrington J.M., Millar S.R., Perry I.J., Phillips C.M. (2023). Plant-Based Dietary Indices and Biomarkers of Chronic Low-Grade Inflammation: A Cross-Sectional Analysis of Adults in Ireland. Eur. J. Nutr..

[57] Huang Y., Li X., Zhang T., Zeng X., Li M., Li H., Yang H., Zhang C., Zhou Z., Zhu Y. (2023). Associations of Healthful and Unhealthful Plant-Based Diets with Plasma Markers of Cardiometabolic Risk. Eur. J. Nutr..

[58] Cheng E., Hong C.-C., Ergas I.J., Caan B.J., Kwan M.L., Roh J.M., Cheng T.-Y.D., Sharma N.J., Hanson J.R., Minderman H. (2025). Plant-Based Diet, Inflammation Biomarkers and Body Composition among Women with Breast Cancer: The Pathways Study. Br. J. Nutr..

[59] Barnard N.D., Cohen J., Jenkins D.J.A., Turner-McGrievy G., Gloede L., Green A., Ferdowsian H. (2009). A Low-Fat Vegan Diet and a Conventional Diabetes Diet in the Treatment of Type 2 Diabetes: A Randomized, Controlled, 74-Wk Clinical Trial. Am. J. Clin. Nutr..

[60] Hall K.D., Guo J., Courville A.B., Boring J., Brychta R., Chen K.Y., Darcey V., Forde C.G., Gharib A.M., Gallagher I. (2021). Effect of a Plant-Based, Low-Fat Diet versus an Animal-Based, Ketogenic Diet on Ad Libitum Energy Intake. Nat. Med..

[61] Fatihah F., Ng B.K., Hazwanie H., Norimah A.K., Shanita S.N., Ruzita A.T., Poh B.K. (2015). Development and Validation of a Food Frequency Questionnaire for Dietary Intake Assessment among Multi-Ethnic Primary School-Aged Children. Singapore Med. J..

[62] Rychlik E., Stoś K., Woźniak A., Mojska H. (2025). Normy Żywienia Dla Populacji Polski.

[63] Neufingerl N., Eilander A. (2021). Nutrient Intake and Status in Adults Consuming Plant-Based Diets Compared to Meat-Eaters: A Systematic Review. Nutrients.

[64] Clem J., Barthel B. (2021). A Look at Plant-Based Diets. Mo. Med..

[65] Stahler C. (2005). Vegetarian Resource Group How Many Youth Are Vegetarians? How Many Kids Don’t Eat Meat?. Veg. J..

[66] Kim H., Lee K., Rebholz C.M., Kim J. (2020). Plant-Based Diets and Incident Metabolic Syndrome: Results from a South Korean Prospective Cohort Study. PLoS Med..

[67] Heianza Y., Zhou T., Sun D., Hu F.B., Qi L. (2021). Healthful Plant-Based Dietary Patterns, Genetic Risk of Obesity, and Cardiovascular Risk in the UK Biobank Study. Clin. Nutr. Edinb. Scotl..

[68] Zhuang P., Wang F., Yao J., Liu X., Li Y., Ao Y., Ye H., Wan X., Zhang Y., Jiao J. (2024). Unhealthy Plant-Based Diet Is Associated with a Higher Cardiovascular Disease Risk in Patients with Prediabetes and Diabetes: A Large-Scale Population-Based Study. BMC Med..

[69] Xu X., Yan M., Huo S., Meng S., Yuan C., Wang P. (2025). Plant-Based Diet Index and All-Cause and Cause-specific Mortality: A Prospective Study. Food Funct..

[70] Wang T., Kroeger C.M., Cassidy S., Mitra S., Ribeiro R.V., Jose S., Masedunskas A., Senior A.M., Fontana L. (2023). Vegetarian Dietary Patterns and Cardiometabolic Risk in People With or at High Risk of Cardiovascular Disease: A Systematic Review and Meta-Analysis. JAMA Netw. Open.

[71] Pourreza S., Khademi Z., Mirzababaei A., Yekaninejad M.S., Sadeghniiat-Haghighi K., Naghshi S., Mirzaei K. (2021). Association of Plant-Based Diet Index with Inflammatory Markers and Sleep Quality in Overweight and Obese Female Adults: A Cross-Sectional Study. Int. J. Clin. Pract..

[72] Hillesheim E., Liu W., Yin X., Smith T., Brennan L. (2024). Association of Plant-Based Diet Indexes with the Metabolomic Profile. Sci. Rep..

[73] Austin G., Ferguson J.J.A., Eslick S., Oldmeadow C., Wood L.G., Garg M.L. (2024). Cardiovascular Disease Risk in Individuals Following Plant-Based Dietary Patterns Compared to Regular Meat-Eaters. Nutrients.

[74] Gehring J., Touvier M., Baudry J., Julia C., Buscail C., Srour B., Hercberg S., Péneau S., Kesse-Guyot E., Allès B. (2021). Consumption of Ultra-Processed Foods by Pesco-Vegetarians, Vegetarians, and Vegans: Associations with Duration and Age at Diet Initiation. J. Nutr..

[75] Mulkerrins I., Medin A.C., Groufh-Jacobsen S., Margerison C., Larsson C. (2025). Dietary Intake among Youth Adhering to Vegan, Lacto-Ovo-Vegetarian, Pescatarian or Omnivorous Diets in Sweden. Front. Nutr..

[76] Gan Z.H., Chiu T.H.T., Lin C.-L., Lin M.-N., Kuo P.-H. (2024). Plant-Based Dietary Patterns and Risk of Insomnia: A Prospective Study. Eur. J. Clin. Nutr..

[77] Shin N., Kim J. (2022). Association between Different Types of Plant-Based Diet and Dyslipidaemia in Korean Adults. Br. J. Nutr..

[78] Jindrich C., Joyce J., Daniels E., Procter S.B., Sauer K., Hanson J. (2022). The Nutritional Adequacy and Diet Quality of Vegetarian Menu Substitutions in Urban Kansas Childcare Centers. Nutrients.

