# Supplementary material for: Not All Plants Are Equal: Diet Quality and Inflammation in Vegans and Vegetarians in Urban Poland
Source: Nutrients. 2025 Oct 25;17(21):3361. doi: 10.3390/nu17213361 (PMC12608527; doi:10.3390/nu17213361)
Supplement: Supplementary file 1 [file nutrients-17-03361-s001.zip › nutrients-3926824-supplementary.pdf]

Supplementary material

Table S1. Conversion factors for intake frequencies reported in the Food Frequency Questionnaire (FFQ), adapted from Fatihah et al. (2015) with minor modifications to align with the frequency categories used in the present study.

Frequency of intake	Conv. Factor (formula)
Per day	
1	1.0 (1.02/1)
Per week	
4-6	0.71 (5.0/7)
1-3	0.29 (2.0/7)
Per month	
1-3	0.07 (2.0/30)
<1	0.02 (0.5/30)
Never	
0	0

Table S2. Examples of food items included in each of the 18 food groups.

Food group	Items in the food frequency questionnaire	PDI	hPDI
**Healthy plant food**			
Whole grains	Bread: wholemeal, rye, graham, etc.; Oat flakes; Groats; Brown, wild, red rice; Whole grain pasta; Quinoa; Ready-made breakfast cereals (muesli, granola, breakfast cereals); Wasa bread	Positive scores	Positive scores
Fruits	Seasonal domestic fruits (apples, pears, plums, blueberries, raspberries, etc.); citrus fruits; dried fruits; dates, figs	Positive scores	Positive scores
Vegetables	Avocados; Olives; Vegetables, except potatoes; Sweet potatoes; Mushrooms	Positive scores	Positive scores
Nuts	Nuts; Pumpkin and sunflower seeds; Linseeds; Chia and hemp seeds	Positive scores	Positive scores
Legumes	Beans, peas, broad beans;<br>Lentils, chickpeas, hummus, lentil products; Soybeans, soy products, tofu	Positive scores	Positive scores
Vegetable oils	Rapeseed oil; Sunflower oil; Olive oil; Linseed oil; Coconut oil	Positive scores	Positive scores
Tea & coffee	Black tea; Green, herbal, fruit tea; Coffee	Positive scores	Positive scores
**Less healthy plant food**			
Fruits juices	Fruit juices; Freshly squeezed juices	Positive scores	Reverse scores
Refined grains	Wheat bread; White rice; Wheat pasta; Rice and soy pasta; Rice cakes, corn cakes, corn pancakes; Tortillas; Gluten-free products; Pretzels, crackers, other salty snacks	Positive scores	Reverse scores
Potatoes and salty food group	Potatoes; Chips, puffs, nachos, popcorn; Pickled products; Marinated, preserved, and oil-dried vegetables	Positive scores	Reverse scores
Sugar sweetened and artificially	Sweetened carbonated drinks (Cola, Pepsi, Sprite, etc.); Energy drinks (Redbull, Tiger,	Positive scores	Reverse scores

sweetened beverages	Black, Monster, etc.); Sugar-free light carbonated drinks		
Sweets and desserts	Fruit in syrup; Cakes, cookies, sweet rolls; Bars, wafers, etc.; Milk chocolate, dark chocolate, dark chocolate; Pralines, truffles, chocolate candies; Candies, jellies, marshmallows; Salted, sweetened, caramelized, and roasted nuts; Fruit ice creams, sorbets	Positive scores	Reverse scores
**Animal Food Groups**			
Animal fat	butter; lard	Reverse scores	Reverse scores
Dairy	Full-fat milk; Reduced-fat milk; Lactose-free milk/products; Fermented milk drinks (yogurt, kefir, buttermilk, sour milk); Dairy products with increased protein content (yogurts, protein desserts); Soft cottage cheese (so-called white cheeses); Hard rennet cheeses (so-called yellow cheeses), blue cheeses, processed cheeses, feta cheese, spreadable cheeses; Goat and sheep cheeses; Dairy desserts, milk ice cream; Cream	Reverse scores	Reverse scores
Egg	Boiled eggs; Fried eggs	Reverse scores	Reverse scores
Fish or seafood	Lean fish (pollock, cod, perch, hake, pangasius, trout);<br>Oily fish (salmon, mackerel, sardines, herring, eel, large carp);<br>Seafood (shrimp, crab, mussels, oysters, squid, octopus)	Reverse scores	Reverse scores
Meat	Red meat (beef, pork, veal, bacon);<br>Poultry (chicken, duck, turkey);<br>Game (roe deer, wild boar, quail, hare);<br>High-quality cold cuts (ham, tenderloin);<br>Cold cuts and organ meats (e.g., liver, blood sausage, meat, pâtés, headcheese);<br>Sausages	Reverse scores	Reverse scores
Misc. Animal-based foods	Meals from fast-food restaurants (McDonald's, KFC, Burger King);	Reverse scores	Reverse scores

Abbreviations: hPDI – Healthful Plant-based Diet Index; PDI – Overall Plant-based Diet Index
